# Shift in Clinical Profile of Hospitalized Pneumonia in Children in the Non-pharmaceutical Interventions Period During the COVID-19 Pandemic: A Prospective Multicenter Study

**DOI:** 10.3389/fped.2022.782894

**Published:** 2022-03-22

**Authors:** Alexis Rybak, Naïm Ouldali, François Angoulvant, Philippe Minodier, Sandra Biscardi, Fouad Madhi, Isabelle Hau, Audrey Santos, Emilie Bouvy, François Dubos, Alain Martinot, Marie-Aliette Dommergues, Christèle Gras-Le Guen, Elise Launay, Karine Levieux, Ferielle Zenkhri, Irina Craiu, Mathie Lorrot, Yves Gillet, Ellia Mezgueldi, Albert Faye, Stéphane Béchet, Emmanuelle Varon, Robert Cohen, Corinne Levy

**Affiliations:** ^1^ACTIV, Association Clinique et Thérapeutique Infantile du Val-de-Marne, Créteil, France; ^2^Assistance Publique - Hôpitaux de Paris, Pediatric Emergency Department, Robert Debré University Hospital, Université de Paris, Paris, France; ^3^INSERM, Unité Mixte de Recherche 1123 Epidémiologie Clinique et Évaluation Économique Appliquées aux Populations Vulnérables, Université de Paris, Paris, France; ^4^Assistance Publique-Hôpitaux de Paris, Department of General Pediatrics, Pediatric Infectious Disease and Internal Medicine, Robert Debré University Hospital, Université de Paris, Paris, France; ^5^INSERM, Centre de Recherche des Cordeliers, UMRS 1138, Sorbonne Université, Université de Paris, Paris, France; ^6^Groupe de Pathologie Infectieuse Pédiatrique, Paris, France; ^7^Department of Pediatric Emergency, Centre Hospitalier Universitaire Nord, Marseille, France; ^8^Department of Pediatric Emergency, Centre Hospitalier Intercommunal de Créteil, Créteil, France; ^9^Department of General Pediatrics, Centre Hospitalier Intercommunal de Créteil, Créteil, France; ^10^Pediatric Emergency Unit and Infectious Diseases, Université de Lille, Centre Hospitalier Universitaire Lille, Lille, France; ^11^Department of General Pediatrics, Centre Hospitalier de Versailles, Le Chesnay, France; ^12^Department of Pediatrics, Centre Hospitalier Universitaire Nantes, Nantes, France; ^13^Department of Pediatric Emergency, Assistance Publique–Hôpitaux de Paris, Hôpital Le Kremlin-Bicêtre, Université Paris, Paris, France; ^14^Department of General Pediatrics, Assistance Publique–Hôpitaux de Paris, Hôpital Armand Trousseau, Université Sorbonne Paris Cité, Paris, France; ^15^Department of Pediatric Emergency, L'Hôpital Femme Mère Enfant Lyon, Lyon, France; ^16^National Reference Center for Pneumococci, Laboratoire de Microbiologie, Hôpital Intercommunal, Créteil, France; ^17^Paris Est University, IMRB-GRC GEMINI, Créteil, France; ^18^Clinical Research Center, Centre Hospitalier Intercommunal de Créteil, Créteil, France; ^19^Neonates Department, Centre Hospitalier Intercommunal de Créteil, Université Paris Est, Créteil, France

**Keywords:** community-acquired pneumonia, COVID-19, children, non-pharmaceutical intervention, time series analysis

## Abstract

Non-pharmaceutical interventions (NPIs) against coronavirus disease 2019 were implemented in March 2020. These measures were followed by a major impact on viral and non-viral diseases. We aimed to assess the impact of NPI implementation in France on hospitalized community-acquired pneumonia (hCAP) frequency and the clinical and biological characteristics of the remaining cases in children. We performed a quasi-experimental interrupted time-series analysis. Between June 2014 and December 2020, eight pediatric emergency departments throughout France reported prospectively all cases of hCAP in children from age 1 month to 15 years. We estimated the impact on the monthly number of hCAP using segmented linear regression with autoregressive error model. We included 2,972 hCAP cases; 115 occurred during the NPI implementation period. We observed a sharp decrease in the monthly number of hCAP after NPI implementation [−63.0% (95 confidence interval, −86.8 to −39.2%); *p* < 0.001]. Children with hCAP were significantly older during than before the NPI period (median age, 3.9 vs. 2.3 years; *p* < 0.0001), and we observed a higher proportion of low inflammatory marker status (43.5 vs. 33.1%; *p* = 0.02). Furthermore, we observed a trend with a decrease in the proportion of cases with pleural effusion (5.3% during the NPI period vs. 10.9% before the NPI; *p* = 0.06). NPI implementation during the COVID-19 (coronavirus disease 2019) pandemic led not only to a strong decrease in the number of hCAP cases but also a modification in the clinical profile of children affected, which may reflect a change in pathogens involved.

## Introduction

Pneumonia remains a major cause of morbidity and mortality worldwide in children, with 100 million cases and 700,000 deaths each year in children younger than 5 years (1). Numerous pathogens, both viral and bacterial, are involved in pediatric community-acquired pneumonia (CAP), but determining with certainty the causal pathogen remains in most cases a challenge (2). During the 2000s and the 2010s, the role of *Streptococcus pneumoniae* in CAP has been estimated with the “vaccine probe approach” by assessing the impact of the implementation of pneumococcal conjugate vaccines. This approach has shown that *S. pneumoniae* played an underestimated role, especially in hospitalized CAP (hCAP) cases (3, 4). Similarly, several studies showed that implementation of the 13-valent pneumococcal conjugate vaccine (PCV13) led to a major decrease in the number of both pleural effusion and pneumonia cases with high levels of inflammatory markers, whereas the number of pneumonia cases with unelevated levels of inflammatory markers remained unchanged (5). This situation further suggested that pneumonia with high levels of inflammatory markers and pleural effusion may reflect the involvement of *S. pneumoniae* and has been proposed as a key criterion to indicate the need for antibiotic prescription (6). Thus, the clinical profile of CAP may be a proxy to estimate the causal pathogen.

Since March 2020, several non-pharmaceutical interventions (NPIs), such as mask wearing and hand washing, were implemented in France to reduce the spread of severe acute respiratory syndrome coronavirus 2 (SARS-CoV-2) infection. Children were rarely involved in severe coronavirus disease 2019 (COVID-19) cases. This NPI implementation led to an unprecedented change in the epidemiology of numerous viral and bacterial respiratory pathogens, including *S. pneumoniae* infection (7), and a substantial decrease in acute respiratory tract infection. However, the impact of these measures on the clinical profile of hCAP in children remains to be explored.

We aimed to assess the impact of NPI implementation in France on hCAP frequency and the clinical and biological characteristics of the remaining cases.

## Methods

Between June 2014 and December 2020, eight pediatric emergency departments (PEDs) throughout France were asked to report all cases of hCAP in children from age 1 month to 15 years. The eight participating centers accounted for 9% of all annual visits to PEDs in France (3). Cases were defined by the association of fever, a chest radiography finding of a consolidation and/or pleural effusion, and a hospital admission. Data collected included clinical information (age, sex, comorbidities, presence of pleural effusion, antibiotic regimen, and patient outcome), and biological and microbiological results [C-reactive protein (CRP) and procalcitonin (PCT) levels, blood culture, pleural fluid culture, antigen detection test in pleural fluid, polymerase chain reaction (PCR) in pleural fluid, nasopharyngeal PCR, and rapid influenza diagnostic test if performed]. The methodology was detailed previously (3).

The main outcome was the number of hCAP cases over time in children aged 1 month to 15 years. We decided to not use the number of hCAP cases per 1,000 PED visits because measures against the COVID-19 pandemic were associated with a massive change in all PED visits (8). The secondary outcomes were the clinical and bacteriological characteristics of hCAP in the post-NPI era as compared with the pre-NPI period. The status of low and high inflammatory markers was defined for children with available CRP and/or PCT level. hCAP with high inflammatory markers was defined as CRP ≥100 mg/L or PCT ≥5 ng/mL because this threshold is proposed as an indication for immediate antibiotic therapy (6). Conversely, hCAP with low inflammatory markers was defined as CRP <40 mg/L if performed and PCT <0.5 ng/mL if performed. Proven pneumococcal hCAP was defined as a case with a positive blood culture or a positive pleural sample (culture, PCR, and/or antigen test) for *S. pneumoniae*. The same definition was used for *Streptococcus pyogenes* and *Staphylococcus aureus*.

In France, the first lockdown started on March 17, 2020 (8). Thus, we defined the pre-NPI period from June 2014 to March 16, 2020, and the NPI period from March 17, 2020, to December 2020. NPIs implemented in France are detailed at https://www.ecdc.europa.eu/en/publications-data/download-data-response-measures-covid-19.

Vaccination against pneumococcus remained high (>99% of children received at least one dose of PCV13 at age 8 months) during the whole study period, including children born in 2020 (9).

**Figure 1:**
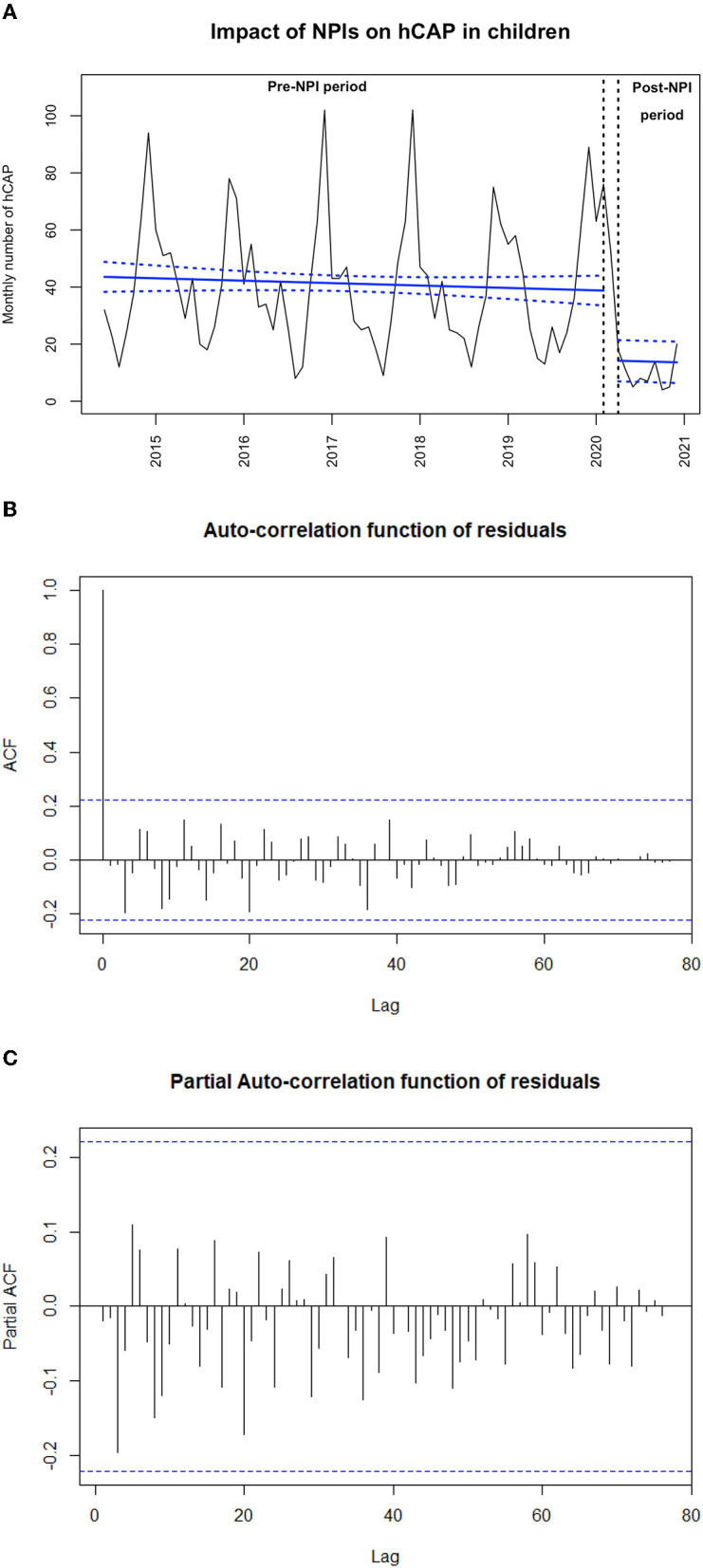
**(A)** Impact of non-pharmaceutical interventions (NPIs) on monthly number of hospitalized community-acquired pneumonia (hCAP) in children from June 2014 to December 2020 (*N* = 2,968). The black line shows the observed data. The bold blue slope shows the model estimates based on observed data (linear regression modeling) with confidence intervals represented as dashed lines. March 16, 2020 (start of the first lockdown), is indicated by the vertical black arrows. **(B)** Auto-correlation function of residuals of the main outcome model. **(C)** Partial auto-correlation function of residuals of the main outcome model.

**Figure 2:**
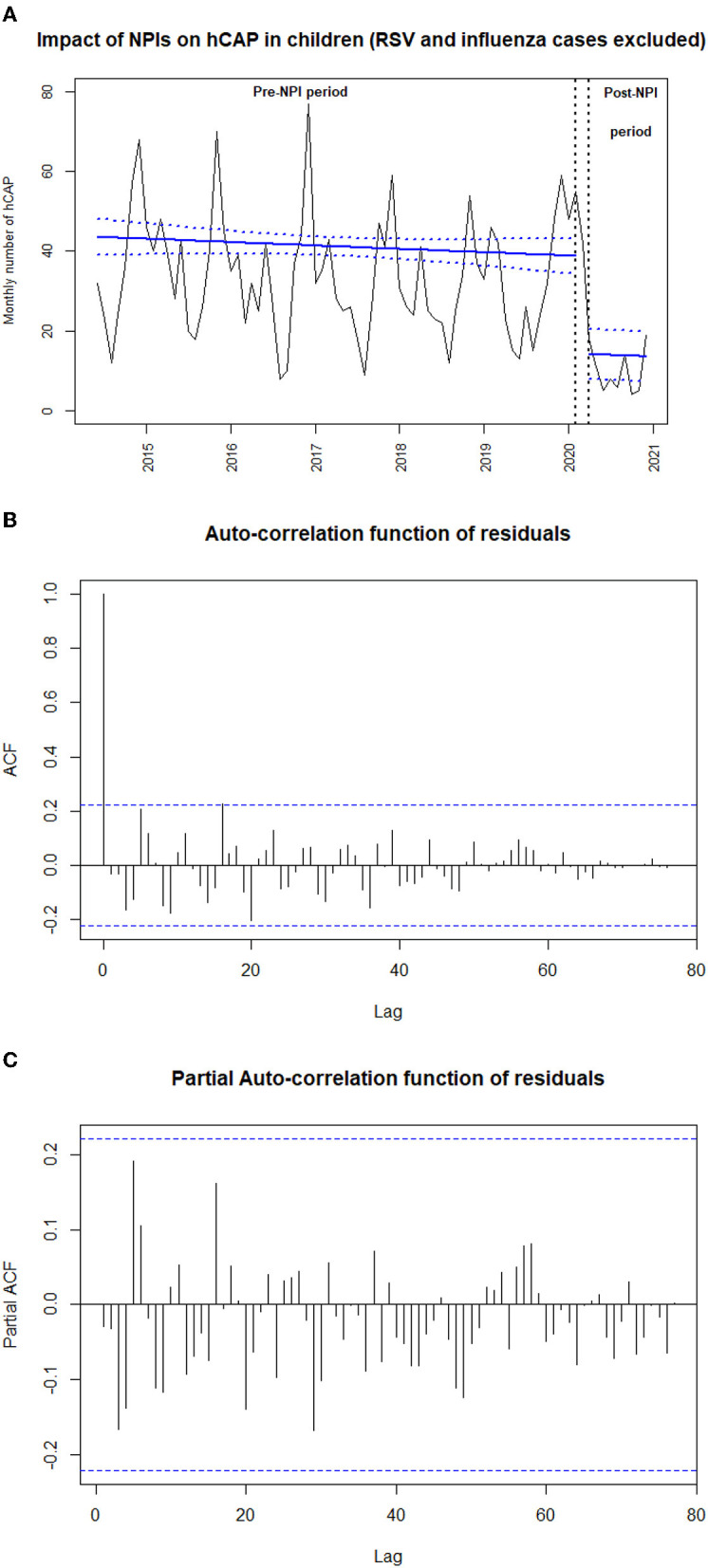
**(A)** Impact of non-pharmaceutical interventions (NPIs) on monthly number of hospitalized community-acquired pneumonia (hCAP) in children after exclusion of respiratory syncytial virus and influenza virus cases from June 2014 to December 2020 (*N* = 2,968). The black line shows the observed data. The bold blue slope shows the model estimates based on observed data (linear regression modeling) with confidence intervals represented as dashed lines. March 16, 2020 (start of the first lockdown) is indicated by the vertical black arrows. **(B)** Auto-correlation function of residuals of the sensitivity analysis. **(C)** Partial auto-correlation function of residuals of the sensitivity analysis.

We used an interrupted time-series analysis approach to assess the change in the number of hCAP cases over time. As described previously, segmented linear regression with autoregressive error was used, with a time unit of 1 month (3). For this analysis, March 2020 was used as the transitional period because NPI implementation occurred during this month. We performed a sensitivity analysis by comparing hCAP prevalence between periods after excluding cases positive for respiratory syncytial virus and influenza virus to avoid seasonality. Furthermore, we estimated the effect on the monthly incidence per 10,000 children younger than 15 years by dividing the monthly number of hCAP by 9% and by using as a denominator the annually age-specific French population (National Institute for Statistics and Economical Studies, https://www.insee.fr/). The validity of the segmented regression models was assessed by visual inspection of correlograms and by residual analysis (Figures 1B,C, 2B,C). Clinical and bacteriological characteristics were compared using χ^2^ and Student *t-*tests between cases before and after the first lockdown. All statistical tests were 2-sided, with *p* < 0.05 considered statistically significant. All statistical analyses involved using R 3.6.3 (R Foundation for Statistical Computing) and STATA 15.1 (StataCorp, College Station, TX, USA).

## Results

From June 2014 to December 2020, we included 2,972 hCAP cases; 115 occurred during the NPI implementation period. Among the hCAP cases, 16.9% had an underlying condition, and 5.6% were hospitalized in a pediatric intensive care unit. Data collected before March 2020 were used to generate a model fitting the observed monthly number of hCAP cases, allowing us to project the number of cases that could have been expected without NPI implementation. We observed a sharp decrease in the number of hCAP cases during NPI implementation [−63.0% (95 confidence interval, −86.8 to −39.2%); *p* < 0.001, Figure 1A]. We found similar results when estimating NPI impact on the monthly number of hCAP cases in children younger than 2 years and in children older than 2 years, on the sensitivity analysis where positive cases for RSV or influenza virus were excluded (Figure 2A), and on the estimated incidence per 10,000 children (Table 1).

**Table 1:** Impact of non-pharmaceutical interventions on hospitalized community-acquired pneumonia in children younger than 15 years old over time.

	**Odds ratio (95% confidence interval)**	***P*-value**
Monthly number of cases (after excluding cases positive for RSV and influenza virus)	−59.1% (−85.0 to −33.3%)	<0.001
Monthly incidence per 10.000 children	−63.1% (−86.6 to −39.7%)	<0.001
Monthly number of cases in children younger than 2 years old	−78.7% (−100 to −45.7%)	<0.001
Monthly number of cases in children older than 2 years old	−54.4% (−80.5 to −28.3%)	<0.001

*hCAP, hospitalized community-acquired pneumonia; NPI, non-pharmaceutical interventions; RSV, respiratory syncytial virus. Percentages are calculated by comparing outcomes in the post-intervention period to expected outcomes without the intervention, based on an estimation with segmented linear regression models*.

Children with hCAP were significantly older during than before the NPI period (median age, 3.9 vs. 2.3 years; *p* < 0.0001), and we observed a higher proportion of low inflammatory marker status (43.5 vs. 33.1%; *p* = 0.02). Furthermore, we observed a trend with a decrease in the proportion of cases with pleural effusion among the remaining hCAP cases (5.3% during the NPI period vs. 10.9% before the NPI; *p* = 0.06). The proportion of hospitalization in pediatric intensive care unit was 5.6% before NPI and 8.8% after (*p* = 0.18). Despite a higher proportion of cases with a blood culture performed, we identified 2 of 115 cases (1.7%) of *S. pneumoniae*, 0 of 115 of *S. pyogenes*, and 0 of 115 of *S. aureus* infection during NPI implementation vs. 62 of 2,857 (2.2%), 44 of 2,857 (1.5%), and 16 of 2,857 (0.5%), respectively, before. Details are in Table 2.

**Table 2:** Characteristics of hospitalized community-acquired pneumonia in children according to the period the before and during the non-pharmaceutical interventions (NPIs) during the COVID-19 pandemic (*n* = 2,972).

	**Before the NPI period (from June 2014, to March 16, 2020) (*n* = 2,857)**	**During NPI period (from March 17, 2020, to December 2020) (*n* = 115)**	***P*-value**
Monthly number of hCAP^*^	41.2	13.9	<0.001
Monthly incidence of hCAP per 10,000 children^*^	3.7	1.3	<0.001
Monthly number of hCAP cases associated with pleural effusion^*^	4.4	1.1	0.002
Male	1,510/2,833 (53.3%)	64/115 (55.6%)	0.62
Age (years) median (interquartile range)	2.3 (1.1–4.7)	3.9 (1.8–8.9)	<0.0001
Apyrexia after 48 h	1,381/1,812 (76.2%)	70/95 (73.7%)	0.57
Underlying condition	462/2,768 (16.7%)	25/112 (21.4%)	0.12
Pleural effusion	308/2,832 (10.9%)	6/113 (5.3%)	0.06
Virus testing	1,237/2,818 (43.9%)	87/110 (79.1%)	<0.0001
Blood culture performed	1,587/2,840 (55.9%)	79/114 (69.3%)	0.005
CRP value available	2,401/2,857 (84.0%)	108/115 (93.9%)	0.004
PCT value available	749/2,857 (26.2%)	78/115 (32.2%)	0.15
Pleural puncture performed	136/2,825 (4.8%)	2/113 (1.8%)	0.13
Low inflammatory markers (CRP <40 mg/L if performed and PCT <0.5 ng/mL if performed)	797/2,404 (33.1%)	47/108 (43.5%)	0.02
High inflammatory markers (CRP ≥100 mg/L if performed or PCT ≥5 ng/mL if performed)	891/2,404 (37.1%)	33/108 (30.6%)	0.17
**Pathogen identified**			
*Streptococcus pneumoniae* cases	62/2,857 (2.2%)	2/115 (1.7%)	0.75
*Streptococcus pyogenes* cases	44/2,857 (1.5%)	0/115	0.18
*Staphylococcus aureus* cases	16/2,857 (0.5%)	0/115	0.42
Virus identified (except rhinovirus alone)	683/2,857 (23.9%)	18/115 (15.6%)	0.04
SARS-CoV-2 identified	1/2,857 (<0.1%)^**^	7/115 (8.0%)	<0.0001
Antibiotic prescription	2,582/2,857 (90.4%)	102/115 (88.7%)	0.36
Hospitalization in pediatric intensive care unit	150/2,686 (5.6%)	10/113 (8.8%)	0.18
Death	10/2,843 (0.3%)	2/115 (1.7%)^***^	0.02

* *Estimated with segmented linear regression model*.

** *The patient was positive for SARS-CoV-2 on March 10, 2020*.

*** *The two patients had severe encephalopathy. CRP, C-reactive protein; hCAP, hospitalized community-acquired pneumonia; PCT, procalcitonin; RSV, respiratory syncytial virus*.

## Discussion

In this time-series analysis of 2,972 cases, the number of hCAP cases in children was divided by 3 during NPI implementation. The reduction in hCAP incidence was sustained until the end of the study despite partial relaxation of NPIs and especially school reopening in May 2020 (8). The major decrease in hCAP and hCAP associated with pleural effusion incidence highlights the reduction in transmission of respiratory pathogens following NPI implementation.

Besides the marked reduction in cases, hCAP in the post-NPI period involved older children, with a higher proportion of cases associated with low levels of inflammatory markers and a decreased trend of cases with pleural effusion among the remaining cases. Several hypotheses should be discussed to explain these findings. First, the NPI measures may have lowered the inoculum of pathogenic agents responsible for hCAP. This effect was described for various respiratory pathogens (measles, influenza, and tuberculosis, for example) and suggestive of SARS-CoV-2 infection but has not yet been described for pneumococcus in humans (10, 11). Furthermore, reduced inoculum has not been proven to lead to reduced levels of inflammatory markers in hCAP and to reduce the risk of pleural effusion. Second, the clinical modification may reflect a change in the pathogens involved. hCAP with pleural effusions or with high levels of inflammatory markers more frequently involves bacterial infections, especially due to *S. pneumoniae* (12–14). Furthermore, epidemiological studies reported a major decrease in hCAP with high levels of inflammatory markers following PCV implementations (4, 5). Thus, the trends observed in the remaining cases during the NPI period could reflect a reduction in pneumococcal infections, alone or in association with a virus. This hypothesis is compatible with the major decrease in invasive pneumococcal diseases observed during NPI implementation (7). Viral infections, especially influenza and respiratory syncytial virus, have been identified as risk factors for pneumococcal diseases in children (15, 16) and may facilitate infections with less-invasive serotypes (16). NPI implementation may have decreased the proportion of bacterial hCAP through its major impact on viral circulation (17, 18). Finally, NPI may have changed the pneumococcal carriage (19), which is the prerequisite for disease, and serotype distribution in children. Of note, vaccination against pneumococcus remained high in France during the whole study period (9).

Our study has several limitations. First, the use of viral tests has changed since the COVID-19 pandemic. In the pre-NPI period, the physician may have used viral tests only when a viral infection was highly suspected. Since the COVID-19 pandemic, children admitted to hospital often undergo systematic viral tests including detection of SARS-CoV-2. Details on the tests used with the nasopharyngeal swab were not available for our study. The types of available tests used (multiplex PCR, antigens tests, and direct immunofluorescence tests) may have changed since NPI implementation. Therefore, interpretation of the differences in the isolated viruses is cautioned. Furthermore, the proportion of cases with a CRP value available was higher in the COVID-19 period. We cannot exclude that a change in testing modified the CRP values during the NPI period. Second, the identification of the pathogens responsible for hCAP remains challenging, particularly when viral–bacterium coinfections occur (2), and a high proportion of children from our cohort had no proven causal pathogen. Third, the small number of hCAP in the NPI-period, related to the major impact of NPI, is limiting the comparison with the pre-NPI period. Further studies will be required to confirm the trends that we have observed. Finally, NPI regroups several interventions, which implies several factors related to pathogen, host, and environment. Our study cannot individualize these factors. In conclusion, NPI implementation during the COVID-19 pandemic led not only to a strong decrease in the number of hCAP cases but also a probable shift in the clinical profile of children affected, which may reflect a change in pathogens involved. This could have implications regarding the CAP diagnostic algorithm and therapeutic management.

## Data Availability Statement

The data that support the findings of this study are available from the corresponding author upon reasonable request.

## Ethics Statement

This study was approved by Robert Debré Hospital Ethics Committee and the French National Data Protection Commission. Legal guardians were informed with a written non-opposition form. Written informed consent was not required to participate in this study in accordance with the national legislation and the institutional requirements.

## Author Contributions

Substantial contributions to the study, including: CL, RC, SBé, and EV: conceptualization/design. CL and RC: funding acquisition. CL, RC, and SBé: methodology. SBé: data curation. AR, PM, SBi, FM, IH, AS, EB, FD, AM, M-AD, CG-L, EL, KL, FZ, IC, ML, YG, EM, and AF: investigation. AR: formal analysis. NO, FA, RC, and CL: supervision/oversight. Participation in the writing and/or revision, including: AR: writing—drafting the initial manuscript. AR, NO, FA, PM, SBi, FM, IH, AS, EB, FD, AM, M-AD, CG-L, EL, FZ, IC, ML, YG, EM, AF, SBé, EV, RC, and CL: writing—review or editing the manuscript, final approval of the version to be published, and agreement to be accountable for all aspects of the work. All authors contributed to the article and approved the submitted version.

## Funding

ACTIV network received funding from Pfizer, the Pediatric infectious Disease Group, and the French National Health Agency for this study. Funders had no role in the design and conduct of the study, collection management, analysis, and interpretation of the data, preparation, review, or approval of the manuscript, and decision to submit the manuscript for publication.

## Conflict of Interest

The authors declare that the research was conducted in the absence of any commercial or financial relationships that could be construed as a potential conflict of interest.

## Publisher's Note

All claims expressed in this article are solely those of the authors and do not necessarily represent those of their affiliated organizations, or those of the publisher, the editors and the reviewers. Any product that may be evaluated in this article, or claim that may be made by its manufacturer, is not guaranteed or endorsed by the publisher.
